# Resolutions of Respect to Dr. H. J. McKellops

**Published:** 1901-06

**Authors:** 


### RESOLUTIONS OF RESPECT TO DR. H. J. McKELLOPS.

At a special meeting of the Society of Dental Science of St. Louis, held Monday evening, April 29, 1901, the following resolutions in memory of Dr. H. J. McKellops were unanimously adopted:

Whereas, In the death of Dr. H. J. McKellops the dental profession has sustained a great loss which will be felt throughout the length and breadth of two continents; and

Whereas, The members of the profession in St. Louis, and especially of this Society, who best knew the depth of his friendship and felt the inspiration of his example, will mourn his loss most deeply; therefore, be it

*Resolved,* That the Society of Dental Science of St. Louis hereby expresses its full appreciation of the valuable services rendered our profession by Dr. McKellops during his long and active life; and

*Resolved,* That in recognition of his distinguished services and the great honor in which he held his profession, a suitable biographical memorial be prepared and framed, with his photograph, and hung in the rooms of this Society; and

*Resolved,* That a copy of these resolutions be sent to the family and to the dental journals, and spread upon the minutes of this Society.

Emma Eames Chase,
Hermann Prinz,
B. L. Lischer,
A. H. Fuller,
*Committee.*

St. Louis, Mo., May 1, 1901.

